# Enhanced Photoluminescence Detection of Immunocomplex Formation by Antibody-Functionalized, Ge-Doped Biosilica from the Diatom *Cyclotella* sp.

**DOI:** 10.3390/nano13131950

**Published:** 2023-06-27

**Authors:** Debra K. Gale, Gregory L. Rorrer

**Affiliations:** School of Chemical, Biological, and Environmental Engineering, Oregon State University, Corvallis, OR 97331, USA; debra.gale@thermofisher.com

**Keywords:** antibody, diatom, frustule, immunocomplex

## Abstract

Diatoms are single-celled algae that biosynthesize cell walls of biogenic silica called “frustules” that are intricately patterned at the submicron- and nanoscale. In this study, we amplified the intrinsic luminescent properties of antibody-functionalized diatom biosilica frustules for enhanced, label-free, photoluminescence (PL) detection of immunocomplex formation. It was hypothesized that metabolically doped GeO centers in antibody-functionalized diatom biosilica would enhance PL emission associated with nucleophilic immunocomplex formation. Germanium (Ge) was metabolically inserted into the frustule biosilica by two-stage cell cultivation of the centric diatom *Cyclotella* sp. The biosilica frustules were isolated by hydrogen peroxide treatment and thermally annealed to convert Ge oxides in the biosilica (0.4 wt% Ge) to luminescent GeO centers. The Ge-doped biosilica frustules were then functionalized with Rabbit Immunoglobulin G (IgG). Upon immunocomplex formation with its complimentary antigen goat anti-Rabbit IgG, the Ge-oxide doped, antibody-functionalized frustule biosilica increased the intensity of PL emission by a factor of 2.6 relative to immunocomplex formation by antibody-functionalized frustule biosilica without Ge. It is proposed that the luminescent GeO centers in the Ge-oxide doped frustule biosilica were more sensitive to radiative recombination than luminescent silanol groups in frustule biosilica without Ge, resulting in a higher PL emission upon immunocomplex formation.

## 1. Introduction

Diatoms are single-celled algae that biosynthesize cell walls of biogenic silica called “frustules” that are intricately patterned at the submicron and nanoscale. The field of “diatom nanotechnology” seeks to harness the unique capability of living diatom cells to fabricate hierarchical biosilica structures with unique properties, and then to impart additional functionality to this nanostructured material to enable a broad range of applications, including photonic and optoelectronic materials [1,2,3,4,5], drug delivery vehicles [6], and biosensing platforms [7,8].

Our initial discovery that frustule biosilica nanostructures created by cultured diatom cells exhibit strong blue photoluminescent (PL) emission in response to excitation by ultraviolet light [9] was leveraged to develop an antibody-functionalized diatom biosensor platform that could detect immunocomplex formation. Specifically, immunocomplex formation with nucleophilic substrates selectively enhanced PL emission [10], whereas immunocomplex formation with electrophilic substrates selectively quenched PL emission [11].

In previous work, we also developed a two-stage cultivation process to metabolically insert germanium dioxide (GeO_2_) [12,13,14] nanophases into the biosilica matrix of the diatom frustule of living diatom cells. Thermal annealing of diatom frustules isolated by hydrogen peroxide treatment of cultured cells converted Ge-oxides, mainly GeO_2_, to GeO luminescent centers with enhanced PL emission [15].

We hypothesize that metabolically doped GeO centers in antibody-functionalized diatom biosilica will also enhance PL emission associated with nucleophilic immunocomplex formation. Toward this end, we fabricated antibody-functionalized, Ge-doped frustule biosilica from the centric diatom *Cyclotella* sp. To dose Ge more precisely into the *Cyclotella* cell, the two-stage cultivation process described earlier [12] was modified from a batch to a fed-batch process.

We report that Ge-doped, diatom frustule biosilica functionalized with the model antibody Rabbit Immunoglobulin G (IgG) enhanced the PL emission intensity upon immunocomplex formation with its complimentary antigen (goat anti-Rabbit IgG) by a factor of 2.6 relative to antibody-functionalized diatom biosilica frustules that did not contain Ge. The diatom cultivation process, diatom biosensor fabrication process, and photoluminescence characteristics are described below.

## 2. Materials and Methods

### 2.1. Two-Stage Diatom Cell Cultivation

Cultures of the photosynthetic, centric marine diatom *Cyclotella* sp. were obtained from the UTEX Culture Collection of Algae (#1269) and maintained on Harrison’s Artificial Seawater Medium as previously described [10]. Diatoms were cultivated in a 4.5 L bubble column photobioreactor [12] at 150 µE m^−1^s^−1^ incident light intensity, 14 h light/10 h dark photoperiod, 0.10 L air L^−1^ culture min^−1^ aeration rate (~350 ppm CO_2_), and 22 °C. A two-stage cultivation strategy for metabolic insertion of Germanium (Ge) oxides into the *Cyclotella* sp. Diatom biosilica frustule was adapted for this present study. The soluble Si and Ge delivery details are provided in Table 1. In Stage I of the cultivation, diatoms were inoculated to an initial cell density of 2.0 × 10^5^ cell/mL in 0.47 mM soluble silicon (Na_2_SiO_3_) and grown to silicon (Si) starvation to achieve stationary phase. In Stage II of the cultivation, at the start of the light phase of the photoperiod, a feed solution consisting of a soluble mixture of 31.3 mM Na_2_SiO_3_ and 0.47 mM GeO_2_ (67:1 mol Si to mol Ge) dissolved in deionized water was continuously delivered into the photobioreactor cell suspension by a syringe pump at a volumetric flow rate of 3 mL h^−1^ for 2 photoperiods (48 h). The cumulative amount of Si delivered in Stage II was designed to provide one cell number doubling after the end of Stage I. A two-stage control cultivation was carried out at the same conditions, where Si without Ge was fed to the cultivation in Stage II. Cell number density was assayed with a Beckman Z2 Coulter Counter, the soluble Si and Ge concentrations in the culture medium were assayed by spectrophotometry, and the weight fraction of Ge in the biosilica frustules was measured by inductively coupled plasma (ICP) spectrometry, as described previously [12].

### 2.2. Frustule Isolation, Thermal Annealing, and Electron Microscopy

*Cyclotella* sp. cell biomass collected at the end of Stage IIB (120 h) was processed for further functionalization studies. Techniques for isolation, thermal annealing, and transmission electron microscopy (TEM) of biosilica frustules described previously [12,15] were used. Briefly, intact frustules were isolated from the diatom cells by treatment with 30 wt% aqueous hydrogen peroxide at pH 2.5 at room temperature for 48 h, which oxidized organic cell material. The frustules were then thermally annealed in air at 400 °C for 2 h. Frustules were suspended in methanol and then deposited onto a holey carbon-coated copper TEM grid, allowed to air dry, and imaged by TEM.

### 2.3. Diatom Frustule Functionalization

Procedures for functionalization of diatom frustule biosilica with Rabbit IgG antibody and anti-Rabbit IgG antigen described previously [10] were used to functionalize Ge-oxide doped *Cyclotella* diatom frustule biosilica. The stoichiometric targets for the functionalization process are summarized in Table 2. Relevant details from this previously published protocol are given below.

**Figure 1:**
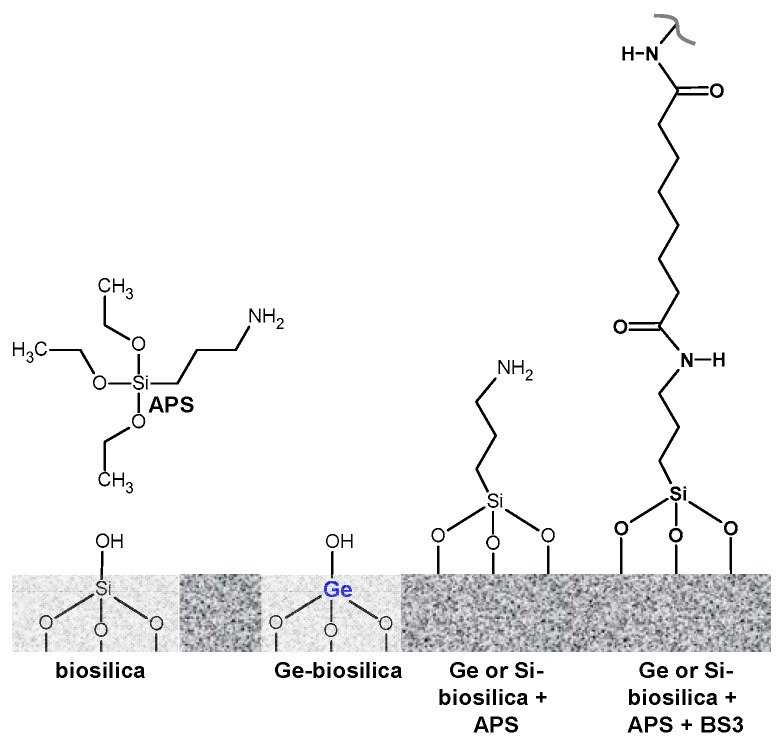
APS functionalization of diatom biosilica containing metabolically inserted Ge.

The diatom frustule surface was functionalized with amine (-NH_2_) groups (Figure 1) by reaction of 10 mg biosilica in 2.0 mL of 2.8 µM 3-aminopropyltrimethoxy-silane (APS) dissolved in ethanol (80 °C, 26 h). The solid (APS-biosilica) was filtered and rinsed in ethanol to remove excess APS and stored in ethanol (10 mg biosilica in 2.0 mL ethanol). An 80 μL aliquot containing 40 μg APS-biosilica was evenly pipetted to diameter of 5 mm on a 18 mm diameter circular glass coverslip. Ethanol was evaporated using air at room temperature, and the biosilica layer remained adhered to the glass cover slip. The APS-functionalized biosilica layers were prepared in triplicate. One APS-biosilica layer was placed in the bottom of a 6-well polystyrene wellplate. Then, 1.8 mL of phosphate buffered saline (PBS, pH 7.2) and 0.200 mL of BS3 solution (0.0657 mg mL^−1^ bis(sulfosuccinimidyl) suberate, Pierce Biotechnology #21580, molecular weight 572.43, corresponding to 0.574 mmol BS3 g^−1^ biosilica) was added to each well and mixed on an orbital shaker at 60 rpm at room temperature for 20 min. BS3 is an amine-reactive crosslinker, but does not add additional amine (-NH_2_) groups to the assembly (Figure 1). The BS3 crosslinker length is 1.14 nm, much smaller than the nominal size of an antibody. The BS3-APS-biosilica films were then dip-rinsed in PBS buffer and transferred to a new well containing 1.8 mL PBS buffer and 0.200 mL Rabbit Immunoglobulin G antibody at 0.0234 g mL^−1^ concentration (Pierce Biotechnology #31235, molecular weight 150,000, 7.8 × 10^−4^ mmol IgG g^−1^ biosilica), and then mixed on an orbital shaker at room temperature for 2 h to complete the attachment of BS3 to an amine group from an amino acid residue on Rabbit IgG. The antibody-functionalized biosilica layer was then dip-rinsed in PBS buffer and stored in buffer before being challenged with its complimentary antigen.

The antibody-functionalized biosilica was challenged with its complimentary antigen, goat anti-Rabbit IgG (Pierce Biotechnology #31210, 150,000 g/mol) in 2 mL PBS at a concentration of 0.0797 mg mL^−1^ (5.3 × 10^−7^ M) under continuous mixing for 60 h on an orbital shaker at room temperature. The antibody–antigen binding constant on functionalized *Cyclotella* frustule biosilica based on label-free PL intensity measurements was 2.83 × 10^−7^ M [10], and so the antigen challenge concentration of 5.3 × 10^−7^ M was about twice the binding constant to ensure a near-saturation response. After the antibody functionalization and antigen binding processes, films were dip-rinsed in PBS buffer to remove excess materials, and then allowed to dry in air at room temperature. Samples prepared for epifluorescence microscopy were functionalized with fluorescein labeled goat anti-Rabbit IgG (Pierce Biotechnology #31635) and imaged under a 470 nm excitation source and GFP filter. All samples were prepared and tested in triplicate.

### 2.4. Photoluminescence Microscopy

A given functionalized biosilica layer deposited on the 10 mm circular glass disk described under Section 2.3 above was mounted with a vertical orientation onto a sample holder. This disk was excited with a 337 nm N_2_ gas laser source (Spectra Physics VSL, 30 kW peak power, 2.4 mW average power, 20 Hz, 4 ns pulse, 120 µJ) as previously described [10]. The laser and detector (Acton Inspectrum 300 spectrometer equipped with CCD detector) were oriented at a 90° angle to one another. The laser beam was cut to a 1 × 3 mm slit and aimed at the vertically oriented, 5 mm biosilica disk to an angle of 45°. The light emitted from the sample surface at a complimentary 45° angle was passed through a 360 nm UV cut-off filter, which removed the reflected 337 nm laser signal. The filtered light signal was then focused to 1.0 mm width. The photoluminescence (PL) spectrum of the focused light emission was collected by the CCD detector using the following settings: 0.20 mm slit width, 300 gratings/mm, 2 s integration time.

### 2.5. Statistical Analysis

All assays for the cultivation process, including cell number density, dissolved Si and Ge concentration, and Ge content in the biomass and diatom biosilica, were conducted in triplicate, with average values and error bars as one standard deviation (1.0 S.D., *n* = 3) reported. Error bars were typically within the size of the data symbol. PL measurements were conducted in triplicate, with replication based on preparation of separate biosilica films detailed in Section 2.3. Furthermore, comparative PL measurements were analyzed by pairwise, single-factor analysis of variance (ANOVA) using Microsoft Excel 2019.

## 3. Results and Discussion

### 3.1. Metabolic Insertion of Ge into Cyclotella Frustule Biosilica

**Figure 2:**
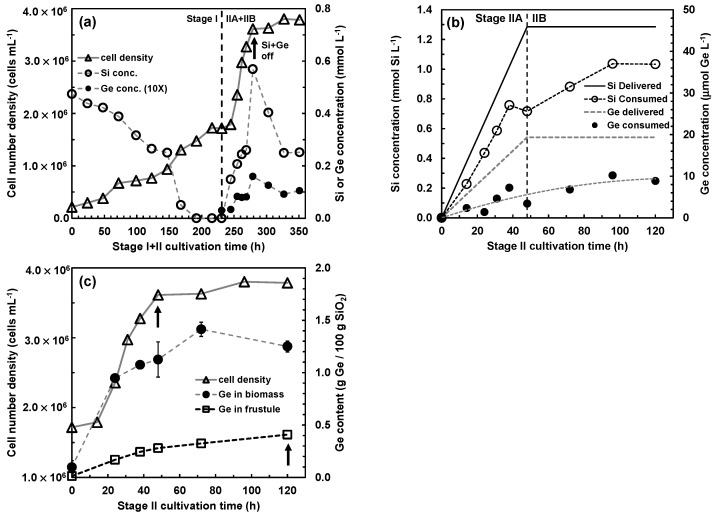
Two-stage cultivation of the centric diatom *Cyclotella* sp. for metabolic insertion of Ge-oxides. (**a**) Cell number density and dissolved Si vs. time profiles; (**b**) Si + Ge delivery and consumption during Stage II; (**c**) Stage II cultivation showing Ge taken up by the biomass, and Ge incorporated into the frustule after biosilica isolation, as wt% of Ge in diatom SiO_2_. The first arrow at 48 h indicates when the Si + Ge feed solution in Stage IIA was turned off, the second arrow at 120 h (end of Stage IIB) indicates the biomass sample used for frustule isolation, functionalization, and PL measurements.

A two-stage cultivation process, with growth of *Cyclotella* cells to silicon starvation in Stage I, followed by fed-batch addition of soluble Si and Ge to silicon-starved cells in Stage II, was used to metabolically insert Ge into the frustule biosilica. The two-stage cultivation of *Cyclotella* sp. diatom cells in the 4.5 L bubble column photobioreactor is presented in Figure 2, using the Si and Ge addition conditions provided in Table 1.

In Stage I of the cultivation process, diatom cells were grown up from 2.14 × 10^5^ to 1.72 × 10^6^ cells/mL (3 doublings). The final cell density was achieved when all soluble silicon was consumed (Figure 2a). The Stage II cultivation process had two parts, IIA and IIB. Fed-batch addition of soluble Si and Ge during Stage IIA slowly dosed in these substrates over a 48 h period to the Si-starved diatom cells generated by Stage I. This process deliberately prolonged the Si starvation state to promote Ge uptake by the diatom cells. As shown in Figure 2b, the relative rates of Si and Ge uptake were comparable. In previous work using a batch uptake process, Ge was taken up at a higher rate than Si because of the surge uptake of Ge [12].

The cumulative amount of Si added to the culture was designed to achieve one cell doubling, which was attained at the end of Stage IIA. In this context, only the daughter valve from cell division was doped with Ge. During Stage IIB, after the Si and Ge feeding was turned off, an additional 72 h allowed further uptake of Ge, presumably during biosynthesis of the girdle band. The Ge content in the biomass, and the Ge content retained in the diatom biosilica frustules after isolation by treatment with hydrogen peroxide, are shown in Figure 2c. At the end of Stage IIB, the cell biomass contained 1.25 ± 0.052 g Ge/100 g biosilica, and the frustules isolated by H_2_O_2_ treatment of the cell biomass contained 0.41 ± 0.030 g Ge/100 g biosilica, indicating that over half of the Ge taken up by the diatom cell leached out during the frustule biosilica isolation process.

**Figure 3:**
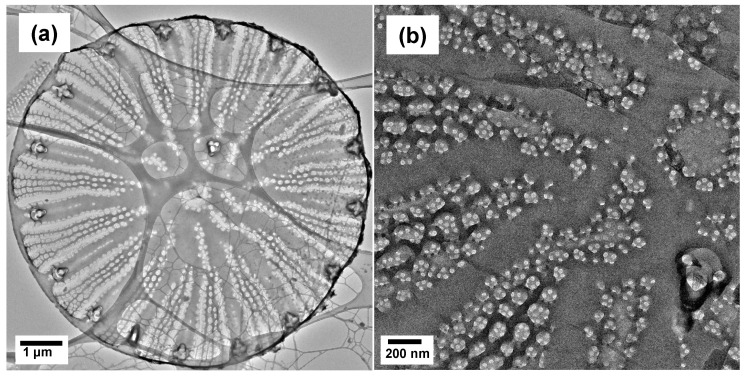

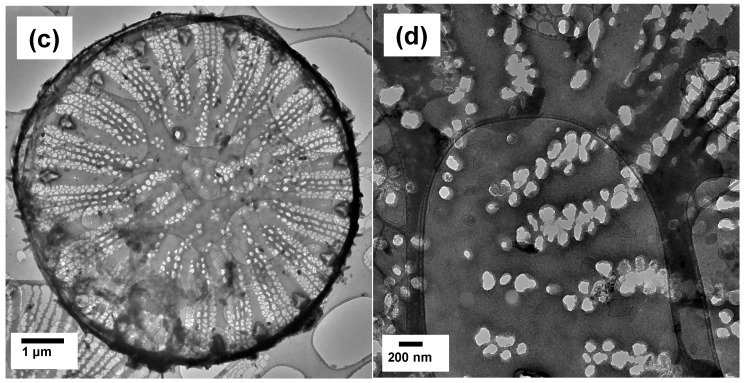
TEM images of *Cyclotella* frustule valve after two-stage photobioreactor cultivation, end of Stage IIB. (**a**,**b**) Control cultivation with no Ge; (**c**,**d**) Cultivation with metabolic insertion of 0.4 wt% Ge in frustule biosilica.

The metabolic insertion of Ge-oxides into the diatom biosilica altered the morphology of the *Cyclotella* frustule valve, as shown in Figure 3. After metabolic insertion of Ge oxide, the biosilica of this centric diatom was densified around the fultoportulae lining the rim of the valve (Figure 3a,c). Furthermore, the fine pore structures disappeared, and the larger pores fused together (Figure 3b,d). Similar changes in fine pore structure were noted previously for metabolic insertion of Ge into the pennate diatom *Pinnularia* [12,13], and the distortion of pore arrays was likely due to lattice mismatch upon substitution of Ge atoms into the silica matrix [16].

### 3.2. Photoluminescence Emission from Ge-Doped, Antibody-Functionalized Cyclotella Frustule Biosilica

**Figure 4:**
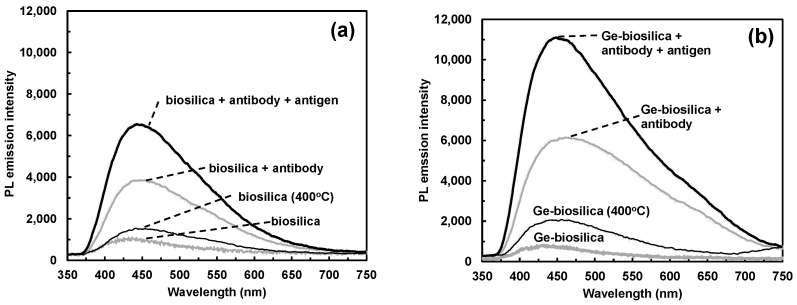
Representative photoluminescence (PL) spectra of functionalized *Cyclotella* diatom frustule biosilica under excitation by 337 nm laser, 2 sec integration time. (**a**) Frustule biosilica with no Ge; (**b**) Frustule biosilica with 0.4 wt% Ge. Biosilica was thermally annealed at 400 °C for 2 h prior to antibody functionalization.

**Figure 5:**
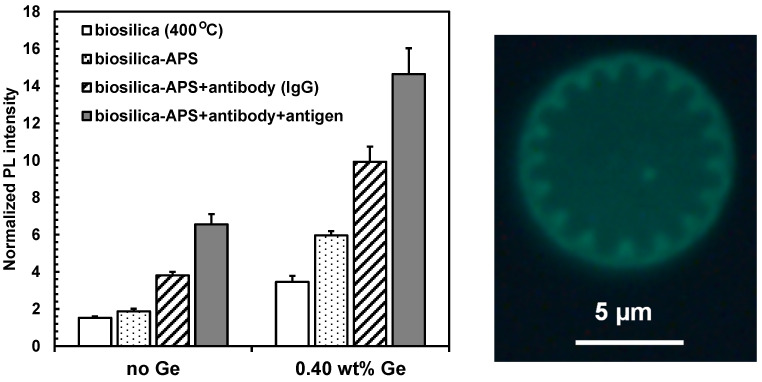
Averaged, normalized peak PL intensity at various stages of functionalization for Cyclotella frustule biosilica vs. biosilica containing 0.4 wt% Ge. All PL emission intensities were normalized to the PL emission of the diatom biosilica frustules isolated from photobioreactor cultured cells (120 h Stage IIB) after hydrogen peroxide treatment, but before thermal annealing. Error bars represent 1.0 standard deviation based on triplicate (*n* = 3) sample preparations. Image on right: Epifluorescence image of Cyclotella frustule biosilica containing 0.4 wt% Ge functionalized with Rabbit IgG antibody and a fluorescein-labeled goat anti-Rabbit IgG antigen.

Amination of *Cyclotella* frustules isolated by hydrogen peroxide treatment, followed by IgG antibody functionalization and then complimentary antigen binding, successively increased the photoluminescence emission intensity in the visible spectrum, as shown in Figure 4. Thermally annealing frustules containing metabolically doped Ge-oxides (0.4 wt% Ge in biosilica) proportionately increased the maximum PL emission intensity at each of these steps by a factor of between 2 and 3, as shown in Figure 5. When diatom biosilica frustules were metabolically doped with Ge-oxides, the sensitivity of antibody–antigen binding (immunocomplex) detection increased by a factor of 2.6 ± 0.1 (1.0 S.D., *n* = 3) relative to frustule biosilica without Ge. However, the peak wavelength, which was centered between 430 and 460 nm at 337 nm laser excitation, did not change after each of these processes.

Although the overall PL emission signal intensity increased, the relative emission increase upon antibody–antigen binding did not change. For antibody-functionalized diatom biosilica without Ge, the relative signal intensity increased before and after antibody-antigen binding was 1.7 ± 0.2, vs. 1.5 ± 0.2 for antibody-functionalized diatom biosilica containing 0.4 wt% Ge. The antibody–antigen binding on Ge-oxide doped *Cyclotella* diatom frustules was verified by epi-fluorescence labeling of the antigen, as shown as the inset in Figure 5.

The PL intensity measurements shown in Figure 5 were statistically analyzed to validate that the differential responses were statistically significant. Specifically, single-factor ANOVA was used to demonstrate if pairwise comparisons were statistically significant at 95% confidence (α = 0.05), as detailed in Table 3. This analysis validated that all possible pairwise comparisons were statistically significant at 95% confidence (*p* < 0.05). Metabolic insertion of Ge into the diatom biosilica enhanced the intrinsic PL emission intensity of the diatom biosilica substrate (BS vs. Ge-BS), antibody functionalization of the biosilica (BS + AB vs. Ge-BS + AB), and immunocomplex formation of the antibody-functionalized biosilica (BS + AB + AG vs. Ge-BS + AB + AG). Furthermore, the PL Ge-doped diatom biosilica PL intensity was enhanced upon antibody functionalization (Ge-BS vs. Ge-BS + AB) and immunocomplex formation (Ge-BS + AB vs. Ge-BS + AB + AG).

Amine groups on organic molecules are known to emit blue photoluminescence [17]. Amination sites that were not functionalized with antibody provided a background PL emission signal that could reduce the selectivity of antibody–antigen binding detection. Therefore, the low concentration of APS in the biosilica amination process was designed to provide a ratio of nominally 6 mol APS/mol bound antibody (Table 2), assuming there were about 3000 IgG molecules per μm^2^ of frustule surface area [10]. Given that there are nominally 80 lysine residues per IgG antibody molecule [18], there were approximately 1 mol -NH_2_ from APS to 12 mol -NH_2_ from IgG.

In our previous study [10], using a biosensing layer consisting of *Cyclotella* frustule biosilica functionalized with the Rabbit IgG antibody, two additional characterization experiments were performed. First, the specificity of binding was validated by challenging the Rabbit IgG functionalized diatom frustule with the non-complimentary antigen goat anti-human IgG, where no net change in PL intensity was observed after 337 nm excitation. Second, the PL intensity was measured as function of the concentration of that complimentary antigen (goat anti-rabbit IgG) from 5.3 × 10^−10^ to 1.6 × 10^−6^ M. From these data, an immunocomplex binding constant (K_m_) of 2.8 × 10^−7^ M and threshold sensitivity at 5.3 × 10^−8^ M (19% of K_m_) were determined. In this present study, the substrate was modified to *Cyclotella* diatom biosilica containing metabolically doped Ge. The antibody (Rabbit IgG), frustule isolation and functionalization protocols, and antigen challenge protocols (goat anti-Rabbit IgG) were not changed from our previous work. Therefore, specificity and sensitivity were not measured again with this system. This study focused solely on the role of Ge-doped diatom frustule biosilica on the PL signal intensity for both antibody functionalization and immunocomplex formation at an antigen concentration where the PL emission signal was saturated. Development of a biosensor platform, which would require assessment of sensitivity, was beyond the scope of this study.

### 3.3. Likely Mechanisms for Enhancement of PL Emission

**Figure 6:**
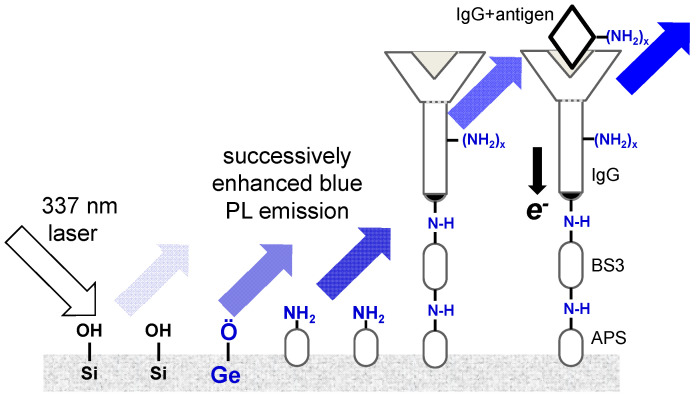
Successive PL emission enhancement of diatom frustule biosilica by metabolic insertion of Ge-oxides, amination of frustule surface, antibody functionalization, and complimentary antigen binding.

The successive PL enhancement processes are summarized in Figure 6. Metabolic insertion of Ge into the *Cyclotella* frustule biosilica, followed by thermal annealing in air at 400 °C for 2 h, enhanced the peak PL emission intensity by a factor of 3.5 (Figure 5). Previously, using X-ray photoelectron spectroscopy (XPS), we showed that Ge-oxide-doped *Pinnularia* biosilica frustules was primarily in the form of GeO_2_. Thermal annealing of Ge-oxide-doped *Pinnularia* frustule biosilica at 400 °C in air for 2 h created strong GeO luminescent centers responsible for enhanced PL emission [15].

Nucleophilic biomolecules absorbed onto photoluminescent diatom frustule surfaces are known to increase PL emission [19]. In previous work [10], we suggested that functionalization of *Cyclotella* frustule biosilica with the nucleophilic antibody Rabbit IgG increased the PL emission of surface silanol (-SiOH) groups by donating electrons to non-radiative PL defect sites, which in turn decreased the probability of non-radiative electron decay and increased the probability of radiative electron decay. Antibody–antigen binding further increased the PL emission intensity of this process, enabling label-free detection of immunocomplex formation. Therefore, we propose that the luminescent GeO centers in the Ge-oxide doped frustule biosilica after thermal annealing were more sensitive to radiative recombination than luminescent silanol groups in frustule biosilica without Ge, resulting in a higher PL emission upon immunocomplex formation.

Time-resolved photoluminescence (TRPL) studies were beyond the scope of this study. However, previous TRPL studies showed that adsorption of nitrous oxide onto photoluminescent diatom biosilica lowered PL emission upon 325 nm laser excitation but did not change fast or slow decay times [20]. From this result, it was inferred that the adsorbed nitrous oxide, an electrophilic molecule, lowered the probability of excitation but did not affect nonradiative decay. In this context, for this present study, the probability of increased radiative electron electronic decay is likely due to the increased probability of excitation due to the presence of nucleophilic antibody and antigen. However, further TRPL studies would be needed to validate this point.

Furthermore, the results of this study are consistent with other recent studies on biomolecule–semiconductor interactions. In a related study [21], enhancement of PL emission under 325 nm UV laser excitation was observed following Aflatoxin B1 (AFB1) binding to photoluminescent, zinc oxide (ZnO) semiconducting nanofibers functionalized with an anti-AFB1 antibody. Energy band diagram analysis revealed that upon AFB1 binding to functionalized ZnO in aqueous solution, the probability of radiative transition responsible for PL emission in the visible range increased, whereas the probability of non-radiative transition decreased, due to the increased separation physical of positive charge carriers associated with the bound AFB1 molecule.

## 4. Conclusions

In this study, we amplified the intrinsic luminescent properties of antibody-functionalized diatom biosilica frustules for enhanced, label-free, photoluminescence detection of immunocomplex formation. This was accomplished by metabolic insertion of Ge oxides into the frustule biosilica by a two-stage cultivation of the centric diatom *Cyclotella* sp., followed by thermal annealing of Ge-oxide doped biosilica (0.4 wt% Ge) to create luminescent GeO centers on the nanostructured frustule. The Ge-oxide doped frustule biosilica increased the intensity of PL emission for immunocomplex formation by a factor of 2.6 relative to frustule biosilica without Ge.

## Figures and Tables

**Table 1:** Two-stage cultivation for metabolic insertion of Ge into *Cyclotella* diatom frustule biosilica.

Cultivation Parameter	Stage I	Stage IIA	Stage IIB	Final
Cultivation time (h)	231	48	72	351
Si conc. beginning of stage (mmol L^−1^)	0.474	0.103	0.404	
Si conc. end of stage (mmol L^−1^)	0.103	0.404	0.252	
Ge conc. beginning of stage (mmol L^−1^)	0.0	3.0	16.0	
Ge conc. end of stage (μmol L^−1^)	0.0	16.0	10.5	
Initial cell number density (cells mL^−1^)	2.14 × 10^5^	1.72 × 10^6^	3.61 × 10^6^	
Final cell number density (cells mL^−1^)	1.72 × 10^6^	3.61 × 10^6^	3.79 × 10^6^	3.79 × 10^6^
Average pH	8.6	9.3	8.8	8.6
Si + Ge feed flowrate (mL feed L^−1^ h^−1^)	0	0.86	0	
Feed solution Si conc. (mM)	0	31.3	0	
Feed solution Ge conc. (mM)	0	0.47	0	
Si delivery rate (mmol Si L^−1^ h^−1^)		0.027		
Ge delivery rate (μmol Ge L^−1^ h^−1^)		0.40		
Total Si added (mmol Si L^−1^)	0.474	1.29	0	1.76
Total Ge added (μmol Ge L^−1^)	0	19.3		19.3

**Table 2:** Target concentrations for functionalization of diatom frustule biosilica.

Substrate and Reactants	Value	Units
Biosilica frustule mass	10.0	mg
Frustule number	1.8 × 10^8^	frustules/mg biosilica
Concentration APS	2.80	μM
Reaction volume	2.0	mL ethanol
Ratio APS/biosilica	5.6 × 10^−4^	μmol APS/mg biosilica
Molar ratio APS/SiO_2_	3.4 × 10^−5^	mol APS/mol SiO_2_
Avogadro’s Number	6.02 × 10^23^	molecules/mol
APS molecules per frustule	1.87 × 10^6^	molecules APS/frustule
Surface density of IgG [10]	~3000	IgG sites/μm^2^ frustule surface
Frustule valve diameter	8.0	μm
IgG sites per frustule	3.0 × 10^5^	IgG sites/frustule surface
APS molecules per IgG site	6	molecules APS/IgG site

**Table 3:** Single-factor ANOVA comparative analysis summary of PL emission data provided in Figure 5. Pairwise comparisons analyzed at 95% confidence (α = 0.05). Abbreviations: BS (diatom biosilica), Ge-BS (Ge-doped diatom biosilica), AB (Rabbit IgG antibody), AG (goat anti-Rabbit IgG antigen).

Pairwise Comparison		*p*-Value	Pairwise Comparison		*p*-Value
BS vs.	Ge-BS	4.4 × 10^−3^			
BS vs.	BS + AB	4.1 × 10^−5^	Ge-BS vs.	Ge-BS + AB	2.2 × 10^−4^
BS vs.	BS + AB + AG	9.5 × 10^−5^	Ge-BS vs.	Ge-BS + AB + AG	1.7 × 10^−4^
BS + AB vs.	BS + AB + AG	1.2 × 10^−3^	Ge-BS + AB vs.	Ge-BS + AB + AG	7.2 × 10^−3^
BS + AB vs.	Ge-BS + AB	1.0 × 10^−3^	Ge-BS + AB + AG vs.	BS + AB + AG	6.2 × 10^−3^

## Data Availability

The data presented in this study are available on request from the corresponding author.
